# Blood-Brain Barrier Integrity and Breast Cancer Metastasis to the Brain

**DOI:** 10.4061/2011/920509

**Published:** 2010-12-29

**Authors:** Farheen Arshad, Lili Wang, Christopher Sy, Shalom Avraham, Hava Karsenty Avraham

**Affiliations:** ^1^Division of Experimental Medicine, Beth Israel Deaconess Medical Center and Harvard Medical School, Harvard Institutes of Medicine, 99 Brookline Avenue 3rd Floor, Boston, MA 02215, USA; ^2^Division of Graduate Medical Sciences, Boston University School of Medicine, Boston, MA 02118, USA

## Abstract

Brain metastasis, an important cause of cancer morbidity and mortality, occurs in at least 30% of patients with breast cancer. A key event of brain metastasis is the migration of cancer cells through the blood-brain barrier (BBB). Although preventing brain metastasis is immensely important for survival, very little is known about the early stage of transmigration and the molecular mechanisms of breast tumor cells penetrating the BBB. The brain endothelium plays an important role in brain metastasis, although the mechanisms are not clear. Brain Microvascular Endothelial Cells (BMECs) are the major cellular constituent of the BBB. BMECs are joined together by intercellular tight junctions (TJs) that are responsible for acquisition of highly selective permeability. Failure of the BBB is a critical event in the development and progression of several diseases that affect the CNS, including brain tumor metastasis development. Here, we have delineated the mechanisms of BBB impairment and breast cancer metastasis to the brain. Understanding the molecular mediators that cause changes in the BBB should lead to better strategies for effective treatment modalities targeted to inhibition of brain tumors.

## 1. Introduction

Breast cancer patients often develop metastatic lesions in the brain [1, 2]. The development of CNS metastasis in patients with solid malignancies represents a turning point in the disease process. The prevalence of CNS metastasis from breast cancer may be increasing due to improved systemic therapy for stage IV breast cancer. The standard treatment for multiple brain lesions remains whole-brain radiation for symptom control, with no improvement in survival. The therapy for a single brain metastasis remains either surgery or radiosurgery, with conflicting information as to the benefit of prior whole-brain radiation. 

To metastasize to the brain, breast cancer cells must attach to microvessel endothelial cells and then invade the blood-brain barrier (BBB), which constitutes the endothelium and the surrounding cells. The BBB is a unique anatomical structure that is mainly defined by tight junctions and adherens junctions between the brain endothelial cells, that strictly regulate the flow of ions, nutrients, and cells into the brain [3, 4]. Compared with endothelial cells from other vascular beds, brain microvascular endothelial cells (BMECs) characteristically have very low permeability to solutes, high electrical resistance, complex tight junctions, and an array of transport systems that both supply the brain with nutrients and eliminates byproducts of brain metabolism. The low permeability is also important in protecting the brain from toxins circulating in the blood and restricting the migration of leukocytes and monocytes. The BMECs form an active permeability barrier and transport system known as the BBB, which is instrumental in the control of the brain fluid milieu. A widely supported hypothesis is that tumor cell adhesion to endothelium induces a retraction of the endothelium, which exposes the vascular basement membrane to the tumor cells. Numerous studies have shown that tumor cells recognize and bind to components in the vascular membrane, thereby initiating extravasation and the beginning of new growth at secondary organ sites. The impairment of the BBB was observed recently in breast cancer patients who developed metastasis to the brain [5].

**Figure 1:**
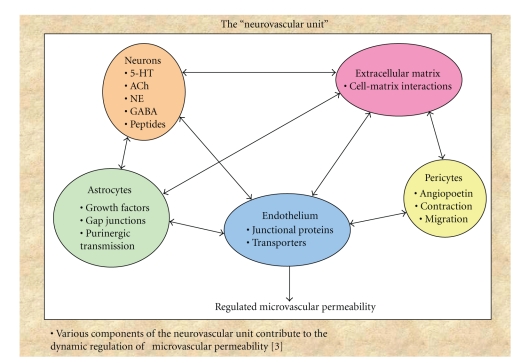


**Figure 2:**
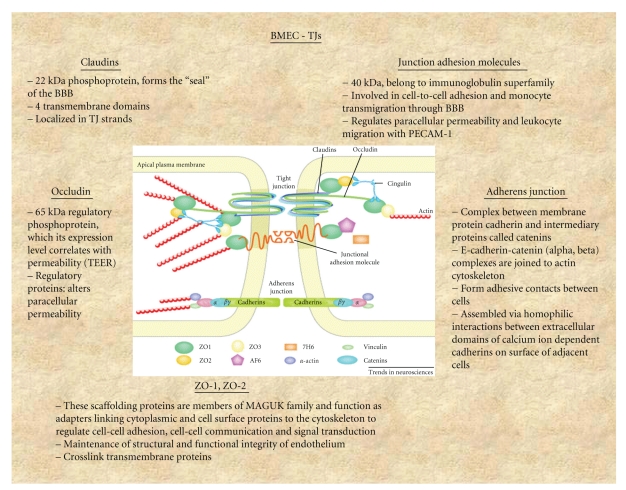
Schematic Presentation of TJs Structures in BMECs [9].

The BBB, a regulated interface between the peripheral circulation and the central nervous system (CNS), is comprised of the cerebral microvascular endothelium, which together with neurons, astrocytes, pericytes, and the extracellular matrix, constitute a “neurovascular unit” (Figure 1) [3, 4, 6]. The BBB is a highly selective diffusion barrier at the level of the cerebral microvascular endothelium, characterized by the presence of mainly tight cell-cell junctions, adherens junctions and lack of fenestrations (Figure 2). The BBB regulates bidirectional control over the passage of a large diversity of regulatory proteins, nutrients and electrolytes, as well as potential neurotoxins [7, 8].

 Increased BBB permeability can be either a consequence of the pathology or a precipitating event [7, 8]. Impairment of the BBB leads to an increase in permeability and formation of edema. Inflammatory mediators such as histamine, bradykinin, and Substance P cause increase in permeability of BBB *in vivo*, which results from the rapid formation of endothelial gaps [7, 8].

## 2. Tight Junctions and Blood-Brain Barrier Integrity

**Figure 3:**
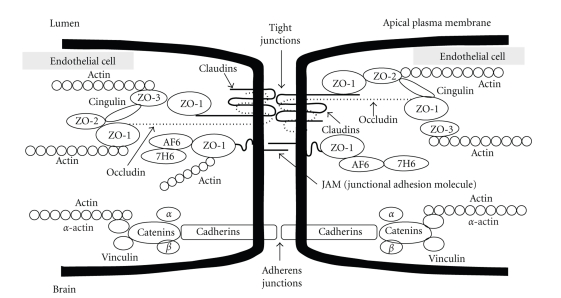
Proposed molecular organization of blood-brain Barrier tight junctions [9].

Most forms of brain injury are associated with BBB disruption, resulting in secondary damage to neural cells. The interendothelial space of the cerebral microvasculature is characterized by the presence of a junctional complex that includes adherens junctions (AJs), tight junctions (TJs) and Gap junctions [8] (see Figure 3). Whereas gap junctions mediate intercellular communication, both AJs and TJs act to restrict the permeability across the endothelium. AJs are ubiquitous in the vasculature and mediate the adhesion of endothelial cells to each other, contact inhibition during vascular growth and remodeling, initiation of cell polarity and partly the regulation of paracellular permeability. The primary component of AJs is VE-cadherin. The *TJs* are the main components that confer the low paracellular permeability and high electrical resistance. TJs are elaborate structures that span the apical region of the intercellular cleft of endothelial barrier tissues. TJs function both as a “zipper” and a “fence” that limit paracellular permeability and are composed of transmembrane proteins as primary seals linked via accessory proteins to the actin cytoskeleton. The TJs are composed of a complex of belt-like zonula occludin, which is localized close to the lumen of the capillary. Electrical resistance *in vivo* across the barrier can increase to approximately 1200 ohm·cm² or higher due to the TJs. The proteins of the TJs include the junctional adhesion molecules (JAM) (JAM-1, JAM-2 and JAM-3), occludin, the claudins, and zonula occludin proteins (ZO-1 and ZO-2). Interestingly, brain microvascular endothelial cells do not express ZO-3 [8]. 

 The ZO proteins are involved in the coordination and clustering of protein complexes to the cell membrane and in the establishment of specialized domains within the membrane [3]. ZO-1 links transmembrane proteins of the TJ to the actin cytoskeleton. The primary cytoskeletal protein, actin, has known binding sites on all ZO proteins and on claudins and occludin. Actin filaments serve both structural and dynamic roles in the cell. ZO-1 binds to actin filaments and to the C-terminus of occludin and claudins, which couples the structural and dynamic properties of perijunctional actin to the paracellular barrier.

The numerous pathways by which specific TJ proteins are regulated and the specific effects of certain pathologies on tight junction (TJ) proteins strongly suggest that therapies targeted to components of the TJ complex and its modulators for the treatment and prevention of breast metastasis to the brain and development of brain tumors are a promising avenue that needs to be explored.

## 3. Genes That Mediate Breast Cancer Metastasis to the Brain

**Figure 4:**
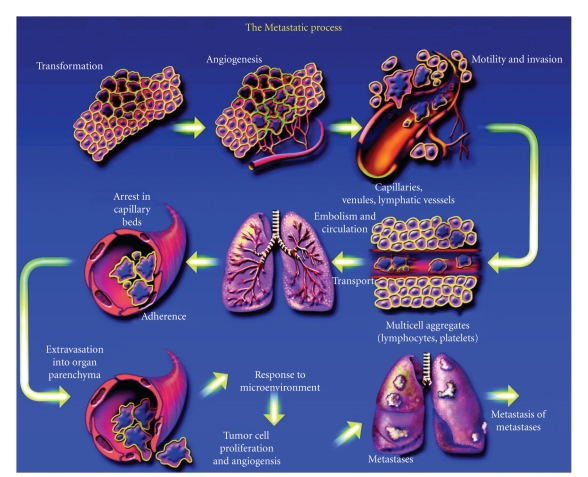
Cancer metastasis. Pathogenesis of cancer metastasis: the process of cancer metastasis consists of sequential, interlinked, and selective steps. The outcome of each step is influenced by the interaction of metastatic cells with homeostatic factors. Each step of the metastatic process is considered rate limiting in that failure of a tumor cell to complete any step effectively terminates the process. Therefore, the formation of clinically relevant metastases represents the survival and growth of unique subpopulations of cells that preexist in primary tumors.

**Figure 5:**
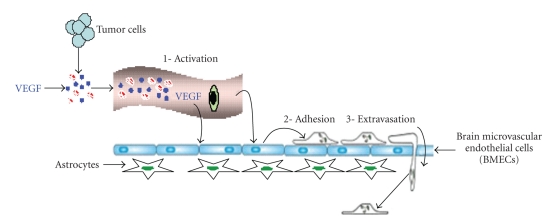
Schematic presentation of tumor cell penetration across the BBB.

The molecular mediators that influence metastasis in distant sites appear to vary by organ (Figure 4). In malignancies of the breast, cancer cells enter a prolonged period of latency before they gain competence to colonize and produce organ-specific metastases [10–12]. During this period of time, disseminated cancer cells may acquire distinct sets of metastasis functions depending on the target organ [13, 14]. Despite the various infiltration and colonization functions, the general process of metastasis can be broken down into local invasion, intravasation, survival in the circulation, extravasation and colonization [15] (Figure 5). After intravasation the cancer cells need to survive in the circulation, travel to specific target organs and extravasate into a microenvironment where they can colonize as secondary tumors [15]. Searches for genetic determinants of metastasis have led to identification of gene signatures that selectively mediate breast cancer cell metastasis to bones, the lungs, and the brain [13–15]. Based on previous work on genomic analysis of breast cancer metastasis to bone and lung, the Massagué group identified three tumor metastasis genes that mediate extravasation through the BBB and cancer cell colonization in the brain [15]. The barriers to metastasis are distinct in organs. To colonize the brain parenchyma, invading tumor cells must penetrate the blood-brain barrier (BBB). Brain capillary walls are more difficult to penetrate due to a tight layer of endothelial cells, tight junctions, and astrocyte foot processes [10, 16]. Functional validation of these genes provided clues as to how cancer cells can penetrate the BBB and initiate tumor growth in brain vasculature. A brain metastasis signature (BrMS) consisting of 17 genes was created using genomic profiling and univariate analysis. The cycloxygenase-2 (COX2), the epidermal growth factor receptor (EGFR) ligand HB-EGF, and the *α*2, 6-sialyltransferase (ST6GALNAC5) were identified as mediators in cancer cell extravasation and infiltration through the BBB. The expression of COX-2 and EGFR ligand HB-EGF enhances the extravasation of cancer cells across the capillaries in an* in vivo *animal model system. The ST6GALNAC5 expression is restricted to the brain both in mice and humans [17]. The knockdown of ST6GALNAC5 reduced the cell passage through a BBB and suppressed metastasis to the brain. In an* in vitro* model of BBB, which consisted of human primary endothelial cells and astrocytes, Massagué and colleagues demonstrated that the ST6GALNAC5 can increase cancer cell adhesion to brain endothelial cells and infiltration through the BBB. The Massagué group has previously identified four lung metastasis gene signature (LMS) that contribute to vascular remodling of tumor blood vessels, entry into the circulation and passage into the lung parenchyma [13]. Comparison of the BrMS with the lung metastasis signature (LMS) showed an overlap of genes between signatures, but not in the bones or liver. Some of the overlapped genes include COX-2, EGFR ligand, ANGPTL4, and LTBP1, which are known to promote disruption of the endothelial barrier and metastasis to the brain and lung. The Massagué group suggested that these genes may specifically contribute to expression signatures that are predictive of metastasis in the brain.

## 4. Cooption of Tumor Cells with Brain Endothelium

Given the observations that certain cancers may have preferential metastatic sites it is natural to investigate what factors, if any, make the brain an “attractive” target for tumor cell growth; specifically in regards to breast primary tumors. The widely accepted “seed and soil” hypothesis first offered by Piaget in 1889 has been credited as the most plausible explanation for the targeted behavior seen in the progression of cancer growths [18]. If accepted, it follows that brain tissue (the “soil”) consisting of neurons, extensive vasculature, and associated neuropil have trophic effects that attract breast primary tumor cells (the “seed”) and facilitate their growth. 

To deduce the validity of this, still prevalent, century-old hypothesis it is vital to observe metastasis before, during, and immediately following successful “colonization” of the distant site. It is within this time frame that any mechanisms, such as Paget's proposal of trophic factors, may play a central role. The time point of interest, referencing current knowledge of metastatic progression, lies in the events between extravasation into distant tissue and any subsequent neoangiogenesis-driven growth (Figure 4) [19]. 

It is important to emphasize at this time that the current discussion will focus on the breast-brain relationship. The genetic heterogeneity of migrating tumor cells is well documented and undoubtedly contributes to profound differences in interaction involving other tissues and organs [20, 21]. Carbonell and colleagues are equally cautious of this distinction, especially in light of their data and its contradiction to the Piagetian “soil” concept. In their paper they reference the relative lack of direct evidence for Paget's hypothesis based on *in vivo* studies [22]. It is this lack of convincing proof that prompted the study of breast cancer cell migration to the brain with a greater focus on the specific steps that lead to successful colonization.

In their paper, direct observation of early tumor colonization revealed a predisposition for growth around existing brain vasculature. This vascular “cooption” contradicts the notion proposed by Paget that trophic factors from distant tissue are responsible for the initial establishment of migrating tumor cells. This is not to deny the possibility that cytokines and chemokines are responsible for drawing tumor cells to certain areas as they traverse the systemic circulation. The possibility of chemoattraction via the CCR7 and CXCR4 receptors has been shown and recognized [23]. Upon arrival, tumor cells preferentially attach to existing blood vessels [22] rather than the chemoattractant releasing neural tissue as expected from the Piagetian viewpoint. Thus, from current knowledge it can be inferred that trophic signaling could play a role in the macroscopic targeting of breast primary tumor cells to the brain, but that the same factors may have a diminished role once access to brain tissue has been attained. They based their initial experiments on the behavior of the MDA-MB-231 cell line. Interestingly, they conducted identical tests with the “brain seeking” MDA231BR cell line as well as A7 (human melanoma) and K1735M2 (murine melanoma) cell lines. All cell lines tested exhibited behaviors consistent with vascular cooption.

The underlying similarity between all conditions and tests is the brain host tissue, its vascular basement membrane and HBMECs. The tight junctions and associated pericytes of the blood brain barrier are a difficult challenge for any invader to penetrate. This includes “invasion” by researchers and clinicians attempting to deliver chemotherapeutic agents and other drugs [24]. The slower rate of extravasation in brain is well noted in comparison to the fenestrated capillaries of other tissues such as bone and liver [25]. It is this unique property of brain microvasculature, a tightly regulated series of junctional complexes that may explain the “antiPiagetian” findings described above. The question becomes whether or not this difficulty in extravasation directly promotes the viability of vascular cooption over direct attachment and growth on neural tissue. It is important to note that these recent findings on vascular cooption within the brain do not diminish the substantial effect of neoangiogenesis on subsequent growth in tumor size and scope. It has been shown that lack of new blood vessel formation and/or remodeling often leads to the death or incapacitation of tumorigenic tissue [26]. Successful migration and initial attachment are steps that must be conceptually separated from the unregulated macroscopic growth that commonly defines cancer. Thus, vascular cooption [22] is the most reliable method by which breast primary tumor cells are able to procure the necessary nutrients and physical scaffolding for initial implantation and growth within the brain.

## 5. Colonization of Tumor Cells around the Blood Vessels

The importance of vascular cooption as a means for tumor cells to survive is highlighted in a study by Gevertz and Torquato [27]. They explain that neoplastic growth is possible even with angiogenesis inhibited as long as vascular cooption is an alternative [27]. Nonetheless, they also report that neoangiogenesis and vascular remodeling is necessary if tumor masses are to grow beyond 1-2mm in diameter. As aforementioned migration and early attachment/colonization should be considered separate from the macroscopic growth step. Clearly, it is the proximity to, as well as early and ongoing interaction with blood vessels in the brain, that contributes significantly to tumor cell fate.

The focus of Gevertz and Torquato on the effects of VEGF, Ang-1, and Ang-2 are interesting in their interplay. They find a pattern of vascular cooption, vessel regression, and robust angiogenesis that requires tight regulation of these factors [27]. The possibility of regulation at the gene level warrants further study. Such a mechanism supports data on the genetic heterogeneity of primary tumor cells and the Darwinian selection of those tumor cells with the capability for metastasis [28]. 

It is known that primary tumors can shed more than a million cells per gram of the tumor mass a day [19]. Despite this constant dispersal of tumor cells, and despite public fear and opinion, metastasis is relatively difficult and inefficient. Thus, the study of physiological changes due to changes at the gene level is a promising direction for cancer research. In light of the importance of vascular cooption and blood vessel colonization to invading tumor cells, a look at gene-regulated factors influencing vascular cooption and colonization could provide a clue to the prevention of secondary growths.

angiogenesis, as we have discussed, is a late event when considering first the chemotaxis of tumor cells, extravasation past the blood brain barrier, and finally successful vascular cooption. The steps preceding angiogenesis, according to Lorger and Felding-Habermann contribute to the lower success rate of brain metastasis compared with other tissues [25]. They report that tumor cells extravasating into brain parenchyma were found to be arrested in G_0_ of the cell cycle. These findings suggest an amount of stress and energy expenditure consistent with a greater effort needed in penetrating the intercellular junctions already discussed in this paper. It is well known that loss of cell attachment proteins and mechanisms leads to the shedding of material from primary tumors [20]. The loss of function in E-cadherin through the disruption of alpha-catenin and/or beta-catenin is well known [1]. We have revealed here that the process for metastasis could very well complete the circle, at least in regards to breast-brain metastasis. Just as loss of adhesion is a necessary first step for tumor cells to leave their primary tissue site, prompt adhesion to the vascular basement membrane of brain endothelial cells is required (and sufficient) for initiation of secondary growth. From evidence collected thus far, it is a possibility that attachment proteins and their constituents largely assume control of primary tumor cell fate as soon as extravasation into brain tissue is complete; wresting control away from any trophic factors. There is evidence that the presence of the blood brain barrier would make such a shift in cellular interaction necessary. Regardless, early colonization around the brain's existing vasculature appears to be necessary for successful metastasis and has the potential for future clinical therapies aimed at prevention of secondary growths within the brain.

## 6. Reactive Astrocytes and Glia on Tumor Growth

The brain provides a unique microenvironment due to its distinctive structure of extracellular matrix (Table 1) and the blood brain barrier (BBB) [29]. It is known that interactions of the host microenvironment and metastatic cells affect the outcome of metastatic progression and tumor survival [30]. Lorger and Felding-Habermann provided in depth *in vivo* analyses of early changes in brain microenvironment upon arrival of breast cancer cells [31]. For studies of the breast cancer cell arrest and extravasation into the brain parenchyma, the Habermann group established breast cancer cell models using MDA-MB-231/brain cells, MDA-MB-435 and murine 4T1 cancer cells. After cell injection into left carotid artery of mice, astrocyte activation was detected in the left hemisphere in brain, showing consistent upregulation in the vicinity of intravascular arrested cancer cells. Reactive astrocytes surrounded and infiltrated brain metastases. Consistent astrocyte activation was detected throughout the extravasation process as well as upregulation of matrix metalloproteinase-9 (MMP-9) proteins in close proximity of extravasating cancer cells. The astrocytic MMP-9 factor can influence cancer cell invasion by promoting growth and angiogenesis in primary brain tumors through release of vascular endothelial growth factors (VEGFs) from the extracellular matrix [32]. In addition to angiogenesis, VEGF also has the function to support the survival and dissemination of breast carcinoma cells [33]. Habermann and Lorger suggested that early involvement of reactive astrocytes may influence the tumor cell fate within the brain parenchyma. In their study, some reactive astrocytes expressed nestin during early cancer cell invasion. In melanoma cells, astrocytes secret haparanase to support the brain microenvironment and the growth of metastatic cells [34], in addition to astrocytes, microglia responses to invading breast cancer cells were detected. Unlike astrocytes, microglia activation associated with the cancer cell brain colonization was not consistent. The active and reactive microglial populations displayed different phagocytic activities and morphology. Despite the differences, a variety of glial responses adds uniqueness to local brain microenvironment of which is essential in determining tumor cell invasion and progression. Astrocytes may have multiple functions in the brain microenvironment. In response to brain injury, astrocytes are activated and recruited to form a glial scar in the site of injury [33]. They can protect neurons from injury induced apoptosis [35]. The Fidler group determined whether reactive astrocytes can also provide neuroprotective properties on protecting tumor cells from cytotoxicity induced by chemotherapeutic drugs. *In vitro* study demonstrated that activated astrocytes protect tumor cells from chemotherapeutic drugs through direct physical contacts.

Astrocytes play important roles in maintaining homeostasis in the brain by regulating nutrient transport, ion trafficking across the extracellular matrix (Table 1) as well as neuronal signaling. It has been shown that specific interactions between brain endothelium and astrocytes within neurovascular units (Figure 1) can influence BBB permeability under pathological conditions. Interactions between the brain endothelium, astrocytes, and neurons may also regulate blood-brain barrier (BBB) function [36]. Cancer cell progression and survival depend on interplay between local host cells and invading tumor cells. Although the specific functions of astrocytes and microglia in early metastatic invasion are yet to be determined, studying local host cells responses during tumor cells invasion may lead to better understanding of tumor microenvironment. Such information could lead to a new avenue of therapeutic targets for brain metastases.

## 7. Angiogenesis and Brain Tumor Growth

New blood vessel formation plays an important role in breast cancer growth, invasion, and metastasis. Tumor growth is preceded by the development of new blood vessels, which provide a pathway for metastasis and nutrients essential for growth. Vascular endothelial growth factor A (VEGF) is a key angiogenic mediator that stimulates endothelial cell proliferation and regulates vascular permeability [37, 38]. Highly proliferative tumors, such as those that are negative for the estrogen, progesterone, and Her2/neu receptors have enhanced angiogenesis that supports rapid growth and early metastasis; also expressing high levels of VEGF [39]. Thus, breast cancer patients that have tumor cells secreting high levels of VEGF may have a higher risk of developing breast cancer metastasis to the brain. VEGF also acts in concert with Angiopoietin2 to regulate vessel growth. In human cancers, increased expression of Ang2 in tumor cells is closely correlated to tumor cell progression, invasiveness, and metastasis [40, 41]. 

 VEGF is essential for angiogenesis and BBB functioning. Our previous studies showed that VEGF upregulated ICAM-1 via phosphatidylinositol 3 OH-kinase/AKT/Nitric oxide pathway and modulated migration of HBMECs [42]. Using human cytokine cDNA array, we found that VEGF-induced significant increase in expression of monocyte chemoattractant protein-1, the chemokine receptor CXCR4 as well as IL-8 in HBMECs [43]. VEGF increased IL-8 production in HBMECs through activation of nuclear factor-*κ*B via calcium and phosphatidylinositol 3-kinase pathways [44]. We also showed that VEGF secreted from breast cancer cells significantly increased the adhesion and penetration of breast cancer cells across the HBMECs monolayer, via changes of VE-cadherin which were inhibited by SU-1498 inhibitor for VEGFR-2 and calcium chelator. VEGF also regulated focal adhesion assembly in HBMECs through activation of FAK and RAFTK/Pyk2 [45]. These focal adhesions are complexes comprised of scaffolding and signaling proteins organized by adhesion to the extracellular matrix (ECM). Further, VEGF upregulated the expression of *α*6 integrin and increased the *α*6*β*1 integrin expression in HBMECs which were important for VEGF induced adhesion and migration as well as in vivo angiogenesis and tumor angiogenesis [46].

VEGF and its cognate receptors are central to the regulation of angiogenesis in both physiological and pathological states. In cancer, local tumor hypoxia stimulates VEGF synthesis and VEGF levels are subsequently elevated in breast cancer. VEGF expression levels correlates with poor prognosis. Blocking of the VEGF-VEGF receptors pathway is accepted as the first antiangiogenic therapy. However, since tumors often develop evasive resistance to this therapy, the development of new antiangiogenic approaches is required for successful antiangiogenic therapy. This can be achieved by better understanding of the receptors and pathways involved in vascular remodeling in brain. Angiopoietins and Tie2 receptor complex were shown to play a critical role in tumor angiogenesis; however their roles in brain BMECs remain elusive.

VEGF is the most important factor in the regulation of the development and differentiation of the vascular system. By acting as a capillary permeability enhancing agent, VEGF also affects the integrity of the BBB. As primary partners of VEGF, angiopoietins (Angs) also play a multiple critical role in vascular development. Angiopoietins are ligands for the Tie 2 receptors and have either agonistic (Ang-1 or Ang-4) or antagonistic (Ang2 and Ang-3) actions regulating vascular survival and expansion. Ang2 is a natural antagonist of Angiogenesis in different microenvironments. Concerted expression of VEGF and Ang2 resulted in increased microvessel density in solid tumors [40]. Ang2 also upregulated MMP-1 and MMP-9 in the presence of VEGF in vitro and MMP elaboration, which participates in the induction of microvessel sprouting in the growing vascular network.

## 8. Clinical Aspects of Breast Cancer Metastasis to the Brain

Brain metastasis, is a significant cause of morbidity and mortality in patients with breast cancer. HER-2 positivity is an increasing recognized risk factor for the development of brain metastasis [47]. Other than Her2 overexpression, there are other factors that increase the risk for breast cancer metastasis to the brain such as negative estrogen and progesterone receptor status, young age, large tumor size, elevated Lactate dehydrogenase (LDH), grading, and number of positive lymph nodes [48].

 As breast cancer is the second most common cause of brain metastasis (after lung cancer) occurring in 10–15% of patients with breast cancer, autopsy studies suggest that the actual incidence is twice (~20 to ~30%) [47]. The incidence of brain metastases is thought to be increasing due to the introduction of more sensitive and accurate diagnostic methods and screening techniques. During the last decade, improved adjuvant and palliative therapy regimens have led to improvement in survival of these patients. In a majority of these patients, the central nervous system dissemination occurs several years (~5 to ~20 years) after systemic lesions have been diagnosed. Approximately 70–80% of the lesions are not solitary but multiple. Cerebrum is the most common site for breast cancer metastasis, following the cerebellum and brainstem [48]. 

Clinically, this parenchymal brain metastasis have an insidious onset with headache (24–48%), neurological deficits as focal motor weakness (16–40%), altered mental status and cognitive dysfunction (24–34%). Seizures, ataxia, nausea, vomiting can also be presenting symptoms. Leptomeningeal metastasis is presented with nonlocalizing symptoms such as headache, nuchal rigidity or cranial neuropathies. 

Brain metastasis can be diagnosed through various techniques. Gadolinium-enhanced magnetic resonance imaging (MRI) is more sensitive than contrast enhanced computed tomography (CT) for identifying both Parenchymal and leptomeningeal disease and is therefore preferred method for detection of brain tumors. Contagious thin axial slices without skips are necessary to pick up small lesions that are missed on CT, especially, in the front-temporal region and in the posterior fossa and brainstem. MRI is also superior in differentiating between solitary and multiple lesions. Approximately 20% of patients thought to have single brain metastases on CT actually have multiple lesions on MRI. Stereotactic brain biopsy must be considered where diagnosis of metastasis is in doubt, especially in patients with a typical presentation as it would lead to change in diagnosis in about 11% of cases. Primary brain tumors, infections, infarction and radiation necrosis are the likely alternative possibilities. 

 Treatment of brain metastasis depends on many factors as such location, number of metastasis, age of the patient, performance, status, and localization of extra cerebral lesions and a prediction of their responses to systemic therapy. On the basis of all these findings, a clinician can decide to have either invasive or noninvasive treatments. Historically, the incidence of clinically appearing CNS metastases in patients with breast cancer is 10–20%. The median time from diagnosis of breast cancer to CNS metastases is about 33 months with 5 months median survival time once diagnosed with cerebral involvement [47]. The majority of cancer patients who develop metastatic brain disease, present with multiple lesions, and death are attributed to uncontrolled metastatic brain disease in approximately 40% of the patients. Median survival in untreated patients with CNS involvement is 1 month; in patients administered with corticosteroids, the survival rate can go to 2 months; and following CNS radiotherapy it can go to 3–6 months. Patients with single CNS lesions and limited systemic disease amenable to surgery or radiotherapy may achieve median survival in the range of 10–16 months. 

 As mentioned earlier, the treatments, prognosis, diagnostic criteria could be different for two types of metastasis, parenchymal and leptomeningeal. The management of patients with brain metastasis can be divided into two groups one for leptomeningeal and other for parenchymal metastasis. Further, there are two approaches for treatment one is symptomatic and the other definitive. Corticosteroids and Anticonvulsants are symptomatic treatments, while the definitive treatment includes whole-brain radiotherapy (WBRT), surgical resection, stereotactic radiotherapy (SRS), whole-brain radiotherapy with radiosensitizers, intracavitary and interstitial brain irradiation, chemotherapy and Chemoradiotherapy.

### 8.1. Leptomeningeal Metastasis

Breast cancer metastasis is the most common cause of metastasis to the leptomeninges, especially from a lobular carcinoma [49]. As described earlier, the symptoms presented are headache, vomiting, ataxia, lethargy, spinal symptoms, cranial nerve palsies and very rarely seizures. Definitive diagnosis is by Cerebrospinal fluid analysis for the presence of malignant cells. Focal radiotherapy is given to symptomatic and bulky sites. The treatment of the entire neuraxis can lead to unacceptable toxicity, mainly leukoencephalopathy and dementia. Those, whose extracranial disease is reasonably controlled, intrathecal chemotherapy can be done through Ommaya reservoir or via lumbar puncture. The most commonly used chemotherapeutic drugs are methotrexate, thiotepa and more recently liposomal cytarabine (Depot Cyt) [50]. The median survival even after multimodality therapy is only 12 weeks.

### 8.2. Parenchymal Metastasis

The most common form of metastasis is thought to be spread via hematogenous route. The management and prevention of CNS metastasis in patients whose tumors over express HER-2/neu need to be reevaluated in the present trastuzumab era, with special consideration for prophylactic cranial irradiation, as trastuzumab is known to increase the incidence of brain metastasis in this group of patients [51–53]. Along with the effectiveness of stereotactic surgery and newer radiotherapy techniques, innovations in blood-brain barrier disruption have expanded the scope of less damaging systemic therapies in brain cancer including metastases [54].

### 8.3. Chemotherapy

The impermeability of BBB to ionized water soluble compounds >180 Da and the presence of the P-glycoprotein efflux pump at the luminal surface of the brain capillaries result in lack of penetration of the chemotherapeutic drugs. Though breast cancer is a chemosensitive disease, there is limited data on the use of chemotherapy for breast cancer metastatic to brain. Most commonly used are cyclophosphamide-based regimen (along with methotrexate, 5FU, prednisolone, etc.), producing response rates 17–61% and median duration of response of 7 months [50]. High dose intravenous methotrexate has resulted in overall response rates of 56% [55]. Recently, temozolamide is being extensively evaluated in phase I and II studies, either alone or in combination with other chemotherapeutic drugs (vinorelbine, cisplatin and capecitabine), for recurrent and progressive brain metastasis from solid tumors, including breast cancer [56, 57]. Theses studies have shown median survival time of 4–7 months.

### 8.4. Whole-Brain Radiotherapy Alone

WBRT is the main stay of treatment for most patients with brain metastasis, which produces symptomatic relief especially of headache and seizures in 75–80% of patients. It also improves survival to about 3–6 months and quality of life and radiological response in up to 60% of the cases [58]. For breast cancer patients, which responds better to WBRT and in patients with longer life expectancy (>6 months), a fraction size of less than 3 Gy is usually administered. Other side effects are alopecia, mild skin toxicity, fatigue, nausea, vomiting, and so forth. Late side effects are urinary incontinence and memory or cognitive disturbances. Late radiation-induced dementia is a rare occurrence, in only 1.9–5.1% of the patients [59].

### 8.5. Surgical Resection

Improved imaging and localization techniques have made surgery an accepted treatment option, particularly in patients with good prognostic factors. There is no direct evidence comparing WBRT alone versus surgery alone. Numerous retrospective studies have reported superiority of surgical resection over WBRT alone, but all of them had inherent selection bias, that is, patients selected for surgery had good performance status, single metastatic lesion, young age and so forth. The median survival in this good prognostic group is approximately 12 months, better than that for WBRT. Further, it has been estimated that only 30% of patients with brain metastases are suitable for surgery.

### 8.6. Stereotactic Radiotherapy

It involves the delivery of a single high-dose fraction of external radiation to a targeted lesion in the brain using multiple cobalt sources (gamma knife), modified linear accelerator (LINAC) or cyber knife. It has a potential to achieve high local control and is essentially used as a substitute for surgical treatment in patients with lesions less than about 3 cm in diameter. The good aspects of SRS are lack of discomfort, minimal invasiveness (no surgical incision), reduced hospitalization time (outpatient basis), with negligible damage to the surrounding healthy tissues. This stereotactic radiotherapy is ideal to target for sterotaxy, being small, spherical, well defined with distinct margins on contrast enhancement. These characteristics help to achieve conformal dose distributions with minimal damage to surrounding tissues. One of its greater advantages is that it can be targeted to those areas where surgical resection is not possible.

Whole-brain radiotherapy with radiosensitizers, intracavitary plus interstitial brain irradiations and chemoradiotherapy are under clinical trial these days and these approaches look promising for future management of brain tumor resulting from breast cancer metastasis.

## 9. Summary

Brain metastasis is a challenging clinical problem and a leading cause of death from cancer. Disruption of the blood-brain barrier was observed in triple-negative breast cancer and basal type breast cancer patients who developed breast cancer metastasis to the brain. Elucidation of the signaling pathways and processes that mediate the early steps of extravasation of breast tumor cells across brain microvascular endothelial cells should provide important information on the biology of tumor cell entry to the brain. Ultimately, this could lead to the design of better therapeutical approaches for blocking changes in permeability and integrity of the brain vasculature and inhibiting brain tumor angiogenesis and tumor growth.

## Figures and Tables

**Table 1:** 

ECM molecules	Candidate or demonstrated receptors	Collagen	Integrins (a1fl1, a2fl1, a3/.3), CD44, syndecan, proteoglycans
Laminins	Integrins (a13, a2fl, a3f3, a6fl, and a7j3; avjIs, a6fl4), dystroglycan, lactose-binding lectins, proteoglycans	Thrombospondins	Integrins avfls, avj3x, axfij, CD36, syndecan, proteoglycans, sulfatides
		Tenascin	Fl 1, integrins (axflj) syndecan, cytotactin binding proteoglycans
Fibronectin	Integrins (av/33, avf36, asfli, a5fl), CD44, syndecan, proteoglycans	Proteoglycans	Hyaluronan, integrins

## Abbreviations

AJs: Adherens junctions
BBB: Blood-brain barrier
BMEC: Brain microvascular endothelial cells
BrMs: Brain metastasis signature
COX-2: Cyclooxygenase 2
CNS: Central nervous system
EC: Endothelial cells
ECM: Extra cellular matrix
EGFR: Endothelial growth factor receptor
FAs: Focal adhesion sites
GAPDH: Glyceraldehyde-3-phosphate dehydrogenase
HUVECs: Human umbilical vein endothelial cells
HBMECs: Human brain microvascular endothelial cells
JAM: Junctional adherens molecules
LCM: Laser capture microdissection
MMPs: Multiple matrix metalloproteinases
TJs: Tight junctions
TEER: Trans-endothelial electrical resistance
VEGF: Vascular endothelial growth factor
VEGFR: Vascular endothelial growth factor receptor.
